# Bone Marrow Plasma Cells Modulate Local Myeloid-Lineage Differentiation via IL-10

**DOI:** 10.3389/fimmu.2019.01183

**Published:** 2019-05-31

**Authors:** Lingzhang Meng, Larissa Nogueira Almeida, Ann-Katrin Clauder, Timo Lindemann, Julia Luther, Christopher Link, Katharina Hofmann, Upasana Kulkarni, David Ming Wong, Jean-Pierre David, Rudolf Armin Manz

**Affiliations:** ^1^Institute for Systemic Inflammation Research, University of Lübeck, Lübeck, Germany; ^2^Institute of Physiological Chemistry and Pathobiochemistry, University of Münster, Münster, Germany; ^3^Department of Osteology and Biomechanics, University Medical Center Hamburg-Eppendorf, Hamburg, Germany; ^4^Department of Internal Medicine, University of Michigan, Ann Arbor, MI, United States

**Keywords:** B cells, plasma cells, IL-10, monocytes, aging

## Abstract

Bone marrow plasma cells have been reported to represent a major source of IL-10; however, the impact of plasma cell derived IL-10 in that tissue remains poorly understood. We confirm in this study that even in the absence of acute immune reactions, mature plasma cells represent the dominant IL-10+ cell population in the bone marrow, and identify myeloid-lineage cells as a main local target for plasma cell derived IL-10. Using Vert-X IL-10 transcriptional reporter mice, we found that more than 50% of all IL-10+ cells in bone marrow were CD138+ plasma cells, while other IL-10+ B lineage cells were nearly absent in this organ. Accordingly, IL-10 was found in the supernatants of short-term cultures of FACS-sorted bone marrow plasma cells, confirming IL-10 secretion from these cells. IL-10+ bone marrow plasma cells showed a B220−/CD19−/MHCII low phenotype suggesting that these cells represent a mature differentiation stage. Approximately 5% of bone marrow leucocytes expressed the IL-10 receptor (IL-10R), most of them being CD115+/Ly6C+/CD11c− monocytes. Compared to littermate controls, young B lineage specific IL-10 KO mice showed increased numbers of CD115+ cells but normal populations of other myeloid cell types in bone marrow. However, at 7 months of age B lineage specific IL-10 KO mice exhibited increased populations of CD115+ myeloid and CD11c+ dendritic cells (DCs), and showed reduced F4/80 expression in this tissue; hence, indicating that bone marrow plasma cells modulate the differentiation of local myeloid lineage cells via IL-10, and that this effect increases with age. The effects of B cell/plasma cell derived IL-10 on the differentiation of CD115+, CD11c+, and F4/80+ myeloid cells were confirmed in co-culture experiments. Together, these data support the idea that IL-10 production is not limited to early plasma cell stages in peripheral tissues but is also an important feature of mature plasma cells in the bone marrow. Moreover, we provide evidence that already under homeostatic conditions in the absence of acute immune reactions, bone marrow plasma cells represent a non-redundant source for IL-10 that modulates local myeloid lineage differentiation. This is particularly relevant in older individuals.

## Introduction

Though most plasma cells are formed in peripheral tissues, the number of plasma cells in the bone marrow steadily increases with age (1). Bone marrow plasma cells are the major source for the production of memory antibodies (2, 3). These cells are preferentially but not exclusively generated during T-dependent immune reactions within germinal centers (4, 5). Compared to plasma cells from other tissues, they exhibit an altered immunophenotype and reduced susceptibility to therapeutic intervention (6–8).

Bone marrow is one of the major primary lymphoid organs after birth, where hematopoietic stem and precursor cells continuously give rise to new lymphoid and myeloid lineage cells (9, 10). A subpopulation of bone marrow myeloid cells expresses CD115, the macrophage colony-stimulating factor (M-CSF) receptor. This population consists of monocytes and “common progenitors of conventional and plasmacytoid dendritic cells” (11), which can further differentiate into macrophages, DCs and osteoclasts (12). CD115+ monocytes/common myeloid progenitors and their progeny exhibit important functions for the maintenance of hematopoietic stem and progenitor cells in the bone marrow, innate and adaptive immunity, wound healing and bone homeostasis. Thereby, these cells are crucial for the outcome of a variety of infectious and inflammatory diseases, such as tuberculosis and atherosclerosis, among others (11, 13–18). Plasmacytoid DCs resemble other bone marrow derived myeloid lineage cells which have a profound capacity to produce inflammatory type 1 interferon, a cytokine of crucial importance for immune protection against viral infection and relevant for the pathogenesis of autoimmune diseases (19).

Factors controlling the expansion and differentiation of bone marrow monocyte/macrophages include M-CSF and the “granulocyte-macrophage colony stimulating factor” (GM-CSF), among others (20–22). Interleukin (IL)-10 is a cytokine with pleiotropic functions (23, 24), which has been reported to promote the maturation of human monocytes into macrophages *in vitro*, while inhibiting their differentiation to DCs (25). Similarly, this cytokine was shown to control monocyte differentiation to macrophages during peritoneal infection in mice (26). Moreover, IL-10 has been reported to restrict the growth of monocyte-derived DCs by the inhibition of cytoprotective autophagy leading to increased apoptosis (27). Several studies indicate that age-related changes in IL-10 production of various cell populations contribute to the age-related chronic progressive increase in the proinflammatory status, sometimes referred to as “inflammaging,” and changes of immune responses in older individuals (28–32).

B cell differentiation into CD138+ plasmablasts *in vitro* is accompanied by the up-regulation of IL-10 production (33). Accordingly, CD138+ plasmablasts/plasma cells represent the major population of IL-10+ cells in the spleen, as demonstrated by using IL-10 transcriptional reporter Vert-X mice (33). Some two decades ago, studies by Simon Fillatreau and David Gray identified B lineage cells as an important source of anti-inflammatory IL-10 in experimental autoimmune encephalomyelitis (34). More recent studies have now revealed that the relevant IL-10+ B lineage cells in this model actually represent CD138+ plasmablasts (35, 36). These plasmablasts were induced during experimental autoimmune encephalomyelitis (EAE) inflammation independent of germinal centers and were selectively found in the draining lymph nodes (36). The same authors demonstrated that these IL-10+ plasmablasts inhibit the activation of pathogenic T cells and thereby control EAE inflammation via modulation of dendritic cell functions. Upon treatment with rituximab, a reagent that selectively depletes B cells and plasmablasts, some multiple sclerosis patients developed increased disease severity, and this effect might be explained by a protective role of B cells/plasmablasts in these patients (37).

As shown by our group, the formation of IL-10+ plasma cells in the spleen can be stimulated by induction of a strong T-dependent reaction when mice are injected with goat-anti mouse IgD. These plasma cells efficiently suppressed the C5a-mediated neutrophil migration and inhibited autoimmune skin inflammation in a model of Epidermolysis bullosa acquisita (38). Furthermore, we found that bone marrow resident murine MOPC315.BM myeloma plasma cells produce IL-10 that mediates increased susceptibility to bacterial infection (38). In aged apolipoprotein E-deficient mice, a model for atherosclerosis, IL-10+ B lineage cells, many of them exhibiting an CD138+ plasma cell phenotype, have been also found within artery tertiary lymphoid organs, i.e., atherosclerosis-associated lymphoid aggregates surrounding the affected arteries (39). During Salmonella infection a novel “regulatory” CD138+ plasma cell population was found that is characterized by the expression of the inhibitory receptor LAG-3+, which following Toll-like receptor stimulation rapidly produces IL-10 (40).

Collectively, these data indicate that following acute immune stimulation, plasmablasts/plasma cells represent an important source of the anti-inflammatory cytokine IL-10, that can dampen autoimmune and infection driven inflammation but can also increase susceptibility to infection. IL-10+/IgM+ bone marrow plasma cells have been shown to be a major local source of IL-10 which may support the formation of immunization induced class-switched plasma cells (41).

In this study, we have confirmed that plasma cells are the dominant source of IL-10 within the bone marrow and have shown that CD115+/Ly6C+ monocytes are a main local target of this cytokine. Furthermore, our data provide evidence that under homeostatic conditions, plasma cell IL-10 is required for normal formation of bone marrow monocytes and DCs in older mice.

## Results

### Plasma Cells Are the Dominant Source of IL-10 in Bone Marrow and CD115+ Myeloid Cells Represent a Major Target

**Figure 1:**
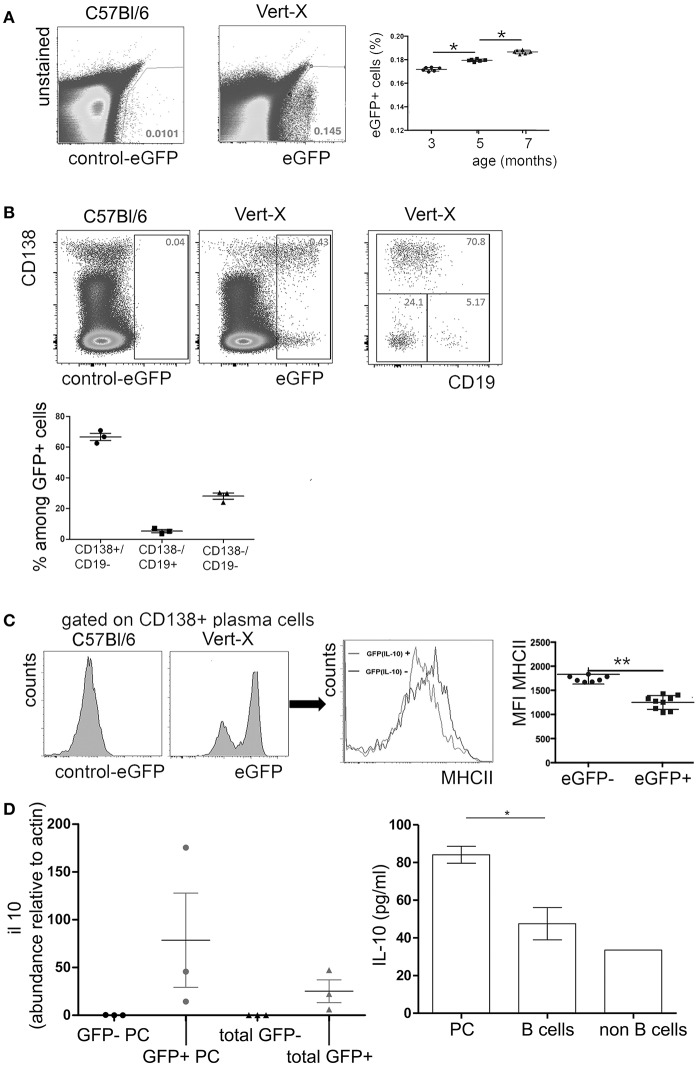
IL-10 expression in murine bone marrow. Single cell suspensions were prepared from femurs and tibia of naïve Vert-X IL-10 transcriptional reporter mice between 3 and 7 months of age. Cells were counterstained for cell type specific markers and analyzed by flow cytometry. **(A)** Representative FACS plots of eGFP (IL-10) expression in Vert-X mice and C57BL/6 controls (left) and frequencies of eGFP (IL-10)+ cells among total bone marrow leucocytes (right). Pooled data from two independent experiments are shown (*n* = 3 for each experiment). **(B)** Gated eGFP (IL-10)+ cells were analyzed for CD19 and CD138 expression (upper row). Frequencies of CD19+/CD138− B cells, CD19−/CD138+ plasma cells and CD19−/CD138− non-B lineage cells among eGFP (IL-10)+ cells (lower plot). Data represents results obtained from one of two independent experiments (*n* = 3) **(C)** Comparison of MHCII expression between eGFP (IL-10)+/CD138+ plasma cells and eGFP (IL-10)−/CD138+ plasma cells. Representative FACS plots and the mean fluorescence intensity (MFI) of MHCII are shown for GFP- and GFP- plasma cells, as indicated. Statistics (Mann-Whitney test). Each dot represents data from a single mouse. Pooled data from three independent experiments are shown (*n* = 3 for each experiment). **(D)** Left: GFP+ plasma cells (GFP+ PC), GFP- plasma cells (GFP- PC), GFP+ non plasma cells (total GFP+), GFP- non plasma cells (total GFP-) from femurs and tibia of Vert-X mice were sorted by FACS and IL-10 expression was determined by real time PCR. Relative il10 mRNA levels compared to actin are shown. Right: Bone marrow plasma cells (PC), B cells, and non B cells from C57/Bl/6 mice were FACS-sorted and cultured in complete RPMI medium plus 20 ng/ml IL-6 for 20 h, when the supernatants were collected and the IL-10 levels were analyzed by ELISA. Data represents results obtained from one experiment (*n* = 3). ^*^P < 0.05, ^**^P < 0.01.

Data from Il10Venus IL-10 reporter mice indicate that bone marrow plasma cells represent a major local source of IL-10 (41). In this study, we analyzed the expression of IL-10 in the bone marrow plasma cell compartment under non-inflammatory steady-state conditions in IL-10 transcriptional reporter (Vert-X) mice. These mice express enhanced green fluorescent protein (eGFP) under the control of the IL-10 promoter. Previous studies showed that eGFP expression of individual cells corresponds well with expression of IL-10 protein in Vert-X mice (33, 38). Under steady-state conditions, ~0.1 to 0.2% of total bone marrow cells were eGFP+ and these frequencies increased with age (Figure 1A). Approximately 60% of the eGFP+ population exhibited a mature CD138+/CD19− plasma cell phenotype (Figure 1B). CD19+/CD138− B lineage cells represented only a very minor fraction of ~5% of the of eGFP+ population, while CD19−/CD138− non-B lineage cells made up for ~30% of eGFP+ cells. eGFP+/CD138+ plasma cells showed reduced expression of MHCII compared to eGFP−/CD138+ plasma cells (Figure 1C) and were mostly B220 negative (data not shown). These results are in accordance with previous findings (5, 41), suggesting that IL-10+ bone marrow plasma cells represent a highly mature differentiation stage.

In accordance with previous findings (33, 38), eGFP expression did correlate well with IL-10 mRNA expression in sorted cells and IL-10 could be also detected in supernatants of sorted bone marrow plasma cells after short-term culture (Figure 1D), indicating that eGFP expression indeed reflects production of IL-10 protein.

**Figure 2:**
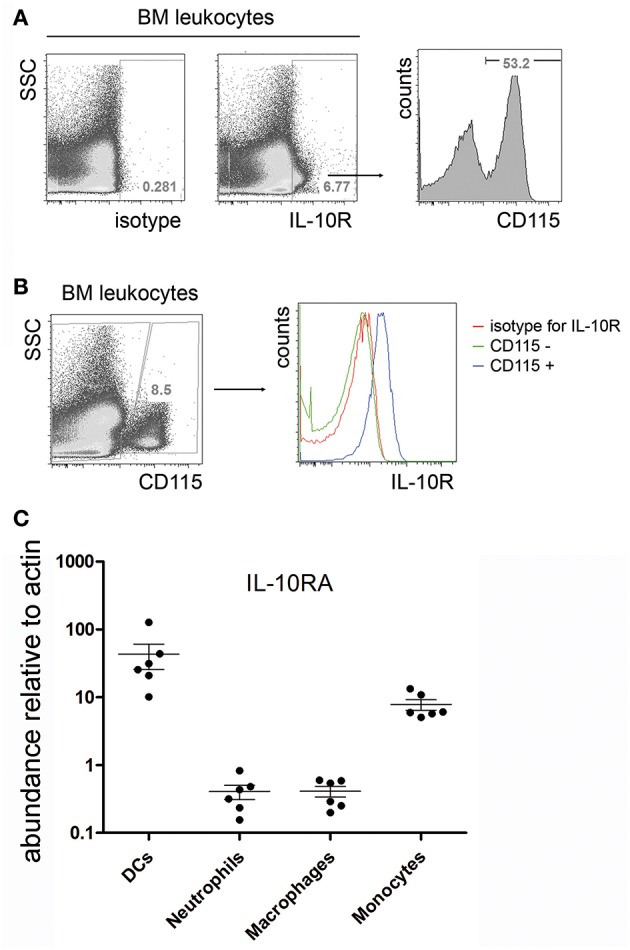
IL-10R expression in the bone marrow. Single cell suspensions from femurs and tibia of C57BL/6 mice were stained for IL-10R and CD115. **(A)** Representative flow cytometric staining for IL-10R compared to an isotype control (left). IL-10R+ cells were electronically gated and analyzed for the expression of CD115 (right). **(B)** IL-10R expression on gated CD115+ bone marrow monocytes. CD3+, B220+, and Gr-1+ cells were excluded from the analysis by electronic gating. Data represents results obtained from one of two independent experiments (*n* = 7). **(C)** Bone marrow dendritic cells (CD115+ Ly6C− CD11c+), neutrophils (CD115− Ly6C− CD11b+ Ly6G+), macrophages (CD115− CD11c− F4/80+), and monocytes (CD115+ Ly6C+ CD11c−) were sorted by FACS and IL-10RA expression was determined by real time PCR. Relative il10ra mRNA levels compared to actin are shown. Pooled data from two independent experiments are shown (*n* = 6).

In order to identify potential local target cells of IL-10, the expression of the IL-10R was investigated. Approximately 4 to 7% of total bone marrow leucocytes expressed IL-10R and the narrow majority of them were CD115+ myeloid lineage cells (Figure 2A), i.e., monocytes and common myeloid progenitors (11). In fact, all CD115+ cells in the bone marrow showed IL-10 receptor expression, as measured by flow cytometry (Figure 2B). Quantification of IL-10RA mRNA by real time PCR of FACS sorted bone marrow subpopulations revealed that CD115+/Ly6C−/CD11c+ DCs and CD115+/Ly6C+/CD11c−monocytes express high levels of mRNA for the IL-10 receptor in comparison to CD115−/Ly6C−/CD11b+/Ly6G+ neutrophils and CD115−/CD11c−/F4/80+ macrophages (Figure 2C).

Together, these data confirm that bone marrow plasma cells represent the dominant source for IL-10 during steady state conditions and indicate that CD115+/Ly6C+/CD11c−monocytes are a potential local target for this cytokine.

### IL-10 From Activated B Cells/Plasma Cells Modulates the Formation of Multiple Myeloid Cell Types *in vitro*

IL-10 derived from plasmablasts in lymph nodes has been shown to alter the maturation of DCs (36). IL-10 is also known to modulate the differentiation of monocytes to macrophages in human cell culture experiments and during peritoneal infection in mice (25, 26). Moreover, IL-10 down-modulates monocyte/macrophage differentiation into osteoclasts (42, 43).

**Figure 3:**
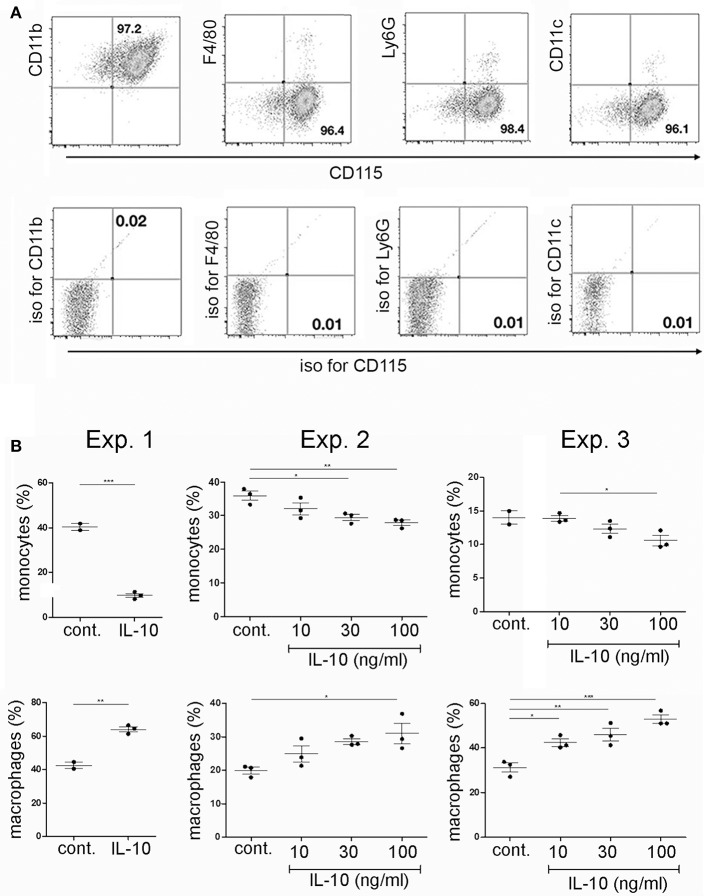
Effects of recombinant IL-10 on monocyte/macrophage differentiation *in vitro*. Primary bone marrow cells were stimulated with M-CSF. After two days of culture, monocytes/common myeloid progenitors were isolated by removal of non-adherent cells. **(A)** Purified adherent cells were analyzed by flow cytometry for the expression of various myeloid markers. **(B)** Purified adherent cells were cultured for another 2 days in the presence of M-CSF and RANKL, with or without addition of various concentrations ofrecombinant IL-10 and subsequently analyzed for the presence of CD115+ and F4/80+ cells. Data from three independent experiments are shown separately, as indicated. Statistics: one-way ANOVA. ^*^P < 0.05, ^**^P < 0.01 and ^***^P < 0.001.

In order to investigate if plasma cells could modulate the differentiation of CD115+ monocytes from bone marrow via IL-10, CD115+ cells were isolated from primary bone marrow cultures by removal of non-adherent cells. Purity of CD115+ cells was >95%. The adherent cells showed a CD115+/CD11b+ F4/80−, Ly6G−, and CD11c− phenotype of monocytes/common myeloid progenitor cells (Figure 3A). These cells were cultured for another 2 days with or without addition of recombinant IL-10 and subsequently analyzed for the expression of CD115+ and F4/80+. Addition of recombinant IL-10 lead to a reduction of the CD115+ populations and an expansion of the F4/80+ populations (Figure 3B).

**Figure 4:**
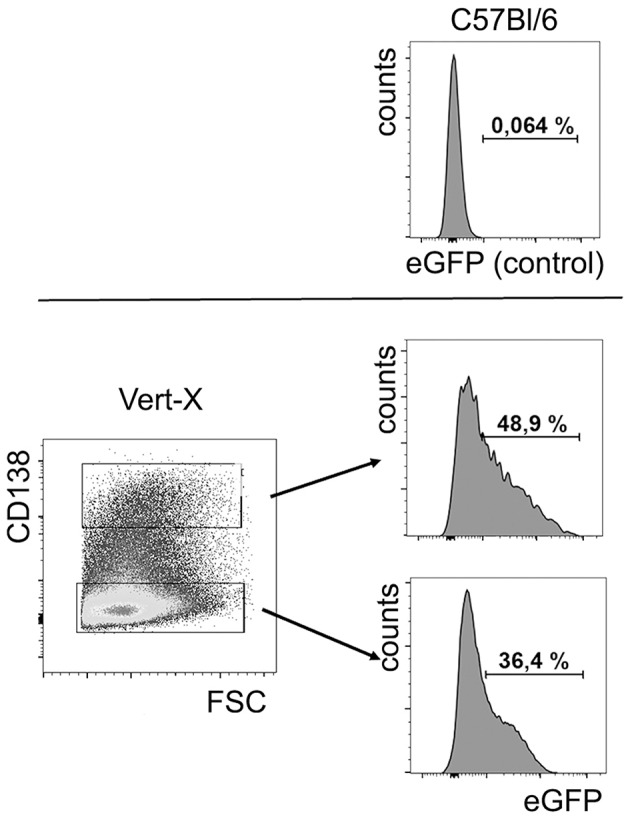
*In vitro* generated plasma cells produce IL-10. B cells were isolated from spleens of IL-10 reporter Vert-X mice and C57BL/6 controls and activated by LPS. After 4 days in culture, IL-10 (eGFP) expression was analyzed in CD138+ plasma cells and CD138- cells. Upper panel: cells from C57BL/6 were used as a negative control for eGFP expression. Lower panel: eGFP expression in CD138+ plasma cells and in CD138− cells, as indicated. Representative FACS data from one of three independent experiments are shown (*n* = 3).

Next, recombinant IL-10 was replaced by IL-10+ B cells/plasma cells. Purified monocytes/common myeloid progenitor cells were supplemented with purified B cells cultured for 4 days with LPS, containing substantial numbers of CD138+ plasma cells/plasmablasts. Approximately 50% of the *in vitro* generated CD138+ plasma cells/plasmablasts in these cultures produced IL-10, as indicated in cultures from IL-10 reporter (Vert-X) mice (Figure 4).

**Figure 5:**
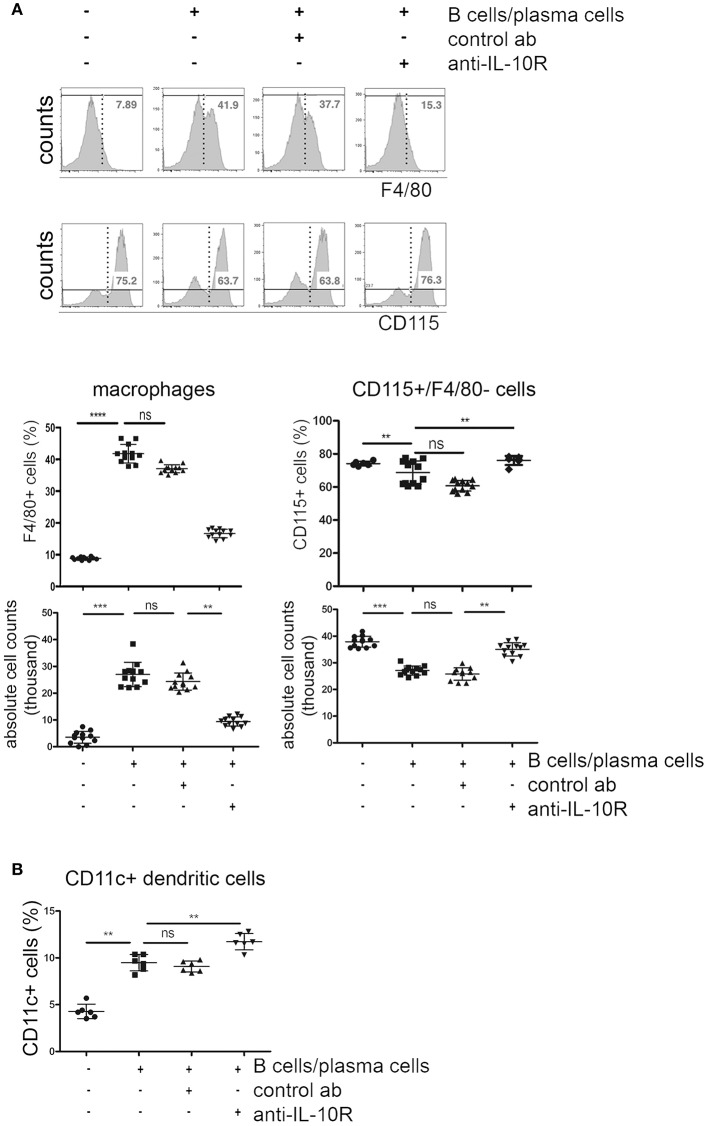
Effects of IL-10+ B cells/plasma cells on myeloid differentiation *in vitro*. Primary bone marrow monocytes were stimulated with M-CSF and RANKL and cultured with or without addition of *in vitro* activated B cells/plasma cells, IL-10R blocking antibodies and control antibodies, as indicated. Three days later, CD115+ monocytes, F4/80+ macrophages, and CD11c+ cells were quantified by flow cytometry. **(A)** Representative FACS plots (upper panel) and statistical analysis (lower panels) of frequencies and absolute numbers of F4/80+ and CD115+/F4/80− cells are shown. Pooled data from four independent experiments are shown (*n* = 3 per experiment). **(B)** Frequencies of CD11c+ cells. Each dot represents the result from one well. Pooled data from two independent experiments are shown (*n* = 3 per experiment). Statistics: one-way ANOVA. ^**^P < 0.01, ^***^P < 0.001 and ^****^P < 0.001.

Comparable to what was observed after addition of recombinant IL-10, co-cultured activated B cells/plasma cells lead to a reduction of ~20% in the frequencies and absolute numbers of CD115+ cells, but to a 4-fold expansion of F4/80+ macrophage populations. Addition of blocking anti-IL-10R antibodies reversed these effects (Figure 5A). F4/80+ cells generated in these cultures showed a CD115+ phenotype. The impact of activated B cells/plasma cells on the formation of CD11c+ DC was less clear. The frequencies of CD11c+ cells increased about 2-fold in cultures where activated B cells/plasma cells were added. Blockade of IL-10 did not reverse this effect (Figure 5B).

These data suggest that activated B cells/plasma cells can promote the formation of CD11c+ cells in an IL-10 independent manner, at least *in vitro*. Though it is known that IL-10 inhibits cytoprotective mechanisms in monocyte derived DC leading to increased apoptosis (27), the mechanism of how activated B cells/plasma cells promote the generation of DC remains to be elucidated.

**Figure 6:**
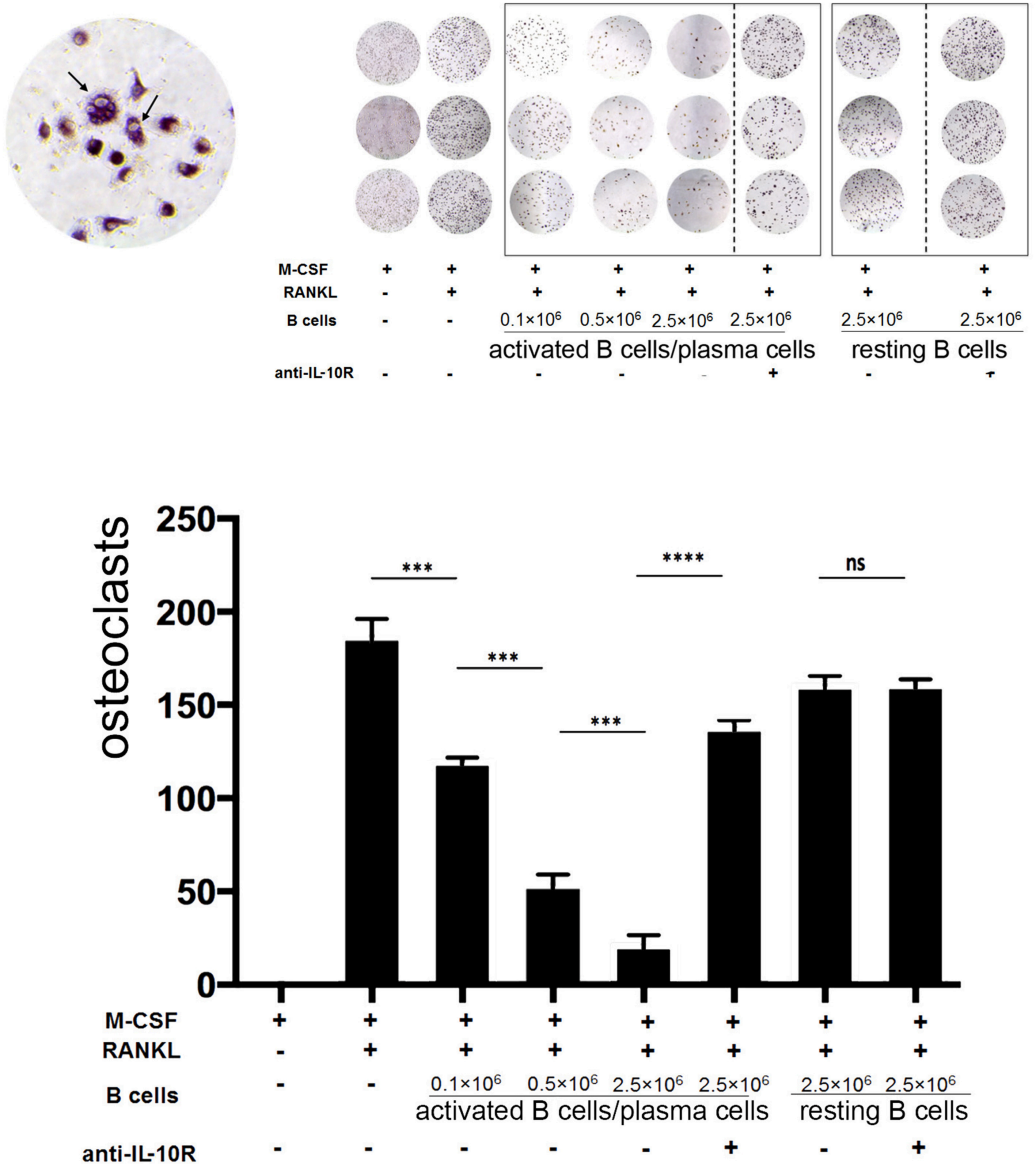
Activated B cells/plasma cells modulate osteoclast differentiation *in vitro* via IL-10. Primary bone marrow monocytes were stimulated with M-CSF and RANKL and cultured with or without addition of *in vitro* activated B cells/plasma cells, naïve B cells, IL-10R blocking antibodies and control antibodies, as indicated. Upper panel: At day 5 to 7, the formation of polynucleated “tartrate-resistant acid phosphatase” positive osteoclast-like cells was observed by microscope. Lower panel: statistical analysis (example: day 7). Statistics: Mann-Whitney test. Data represents results obtained from one of five independent experiments (*n* = 3). ^***^P < 0.001 and ^****^P < 0.0001.

At day 5, the formation of polynucleated “tartrate-resistant acid phosphatase” positive osteoclast-like cells could be observed in the culture by microscope. Addition of activated B cells/plasma cells reduced the formation of osteoclasts up to 8-fold. This effect increased with the numbers of activated B cells/plasma cells added and was dependent on IL-10, as indicated by the following treatment with blocking anti-IL-10R antibodies (Figure 6).

These data demonstrate that activated B cells/plasma cells can modulate the expansion and further differentiation of primary bone marrow CD115+ monocytes/common myeloid progenitors into macrophages, DCs and osteoclast-like cells via IL-10 *in vitro*.

### Bone Marrow Plasma Cell Derived IL-10 Modulates Myeloid Lineage Cells *in vivo* in an Age-Dependent Manner

**Figure 7:**
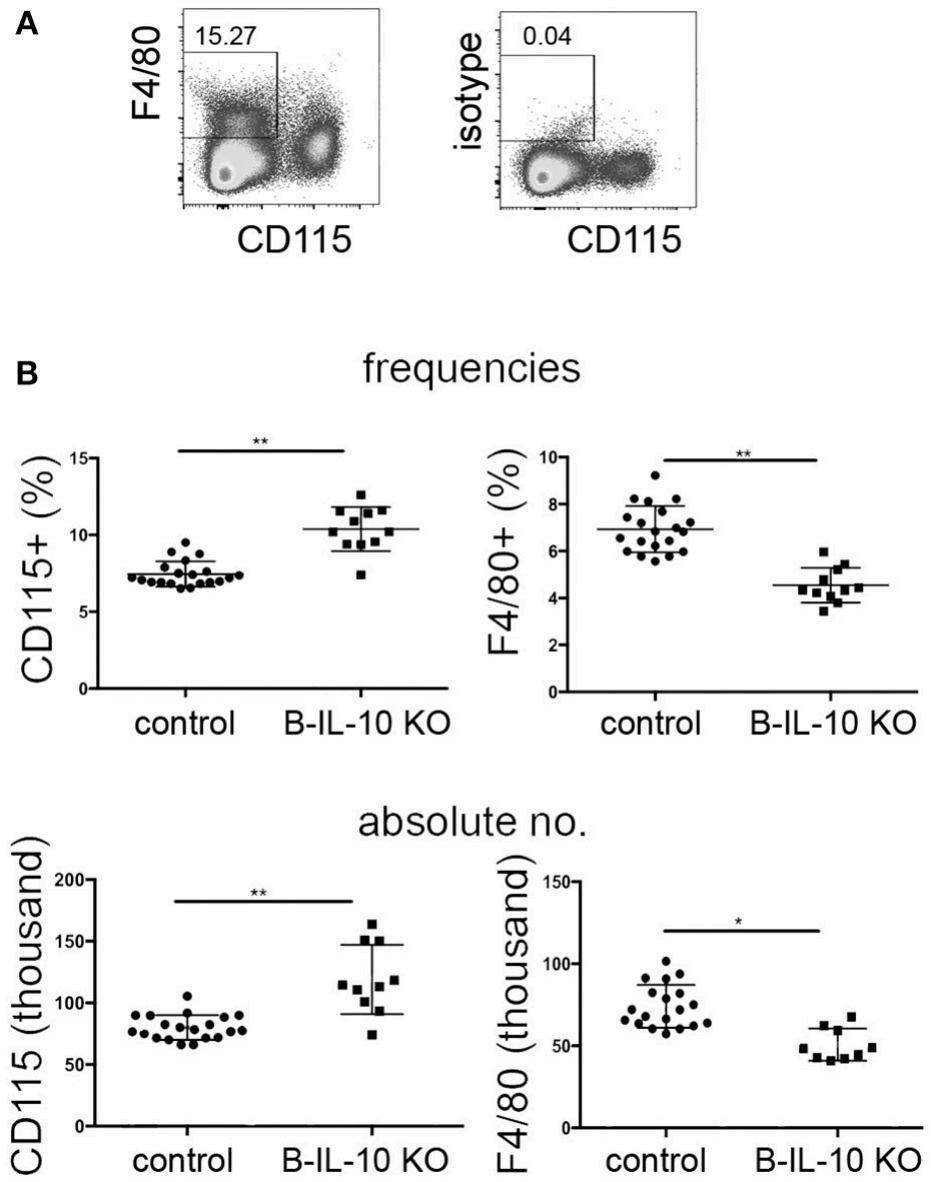
Myeloid differentiation is altered in the bone marrow of B lineage deficient IL-10 KO mice. Single cell suspensions from femurs and tibia of 7 months old B cell specific IL-10 KO mice and their littermate controls were stained for cell lineage markers and analyzed by flow cytometry. **(A)** Representative staining for CD115 and F4/80 are shown. **(B)** Frequencies (upper panel) and absolute numbers (lower panel) of CD115+ and F4/80+ cells in IL-10 KO mice and controls, as indicated. Representative data from one of two experiments are shown. Each dot represents data from a single mouse. Statistics: *t*-test. ^*^P < 0.05 and ^**^P < 0.01.

In order to investigate if B lineage IL-10 could be relevant for the differentiation of myeloid lineage cells *in vivo*, the populations of CD115+, CD11c+, and F4/80+ cells in bone marrow were analyzed in B cell–specific IL-10 knockout mice (CD19 Cre/IL-10 flox/flox) and compared to their littermate controls. Given the fact that CD138+ plasma cells represent the dominant source for IL-10 in this organ while other B lineage cells contribute only minimal to the local production of this cytokine, we expect that these mice represent a suitable model to study the effect of plasma cells derived IL-10 on the local phenotype of bone marrow myeloid cells. Bone marrow plasma cell populations are known to increase with age (1). In our experiments, between the ages of 3 to 7 months the numbers of bone marrow plasma cells increased ~4-fold (Figure S1). At 7 months of age, B cell–specific IL-10 knockout mice exhibited about 20% increased frequencies and numbers of CD115+ bone marrow myeloid cells, while the expression of the macrophage marker F4/80 was reduced compared to their littermate controls (Figures 7A,B). Though we could not identify a distinguished population of F4/80+ cells, F4/80 expression might be an indicator for the presence of a specialized endosteal macrophage subtype in bone marrow, termed osteomacs (44).

**Figure 8:**
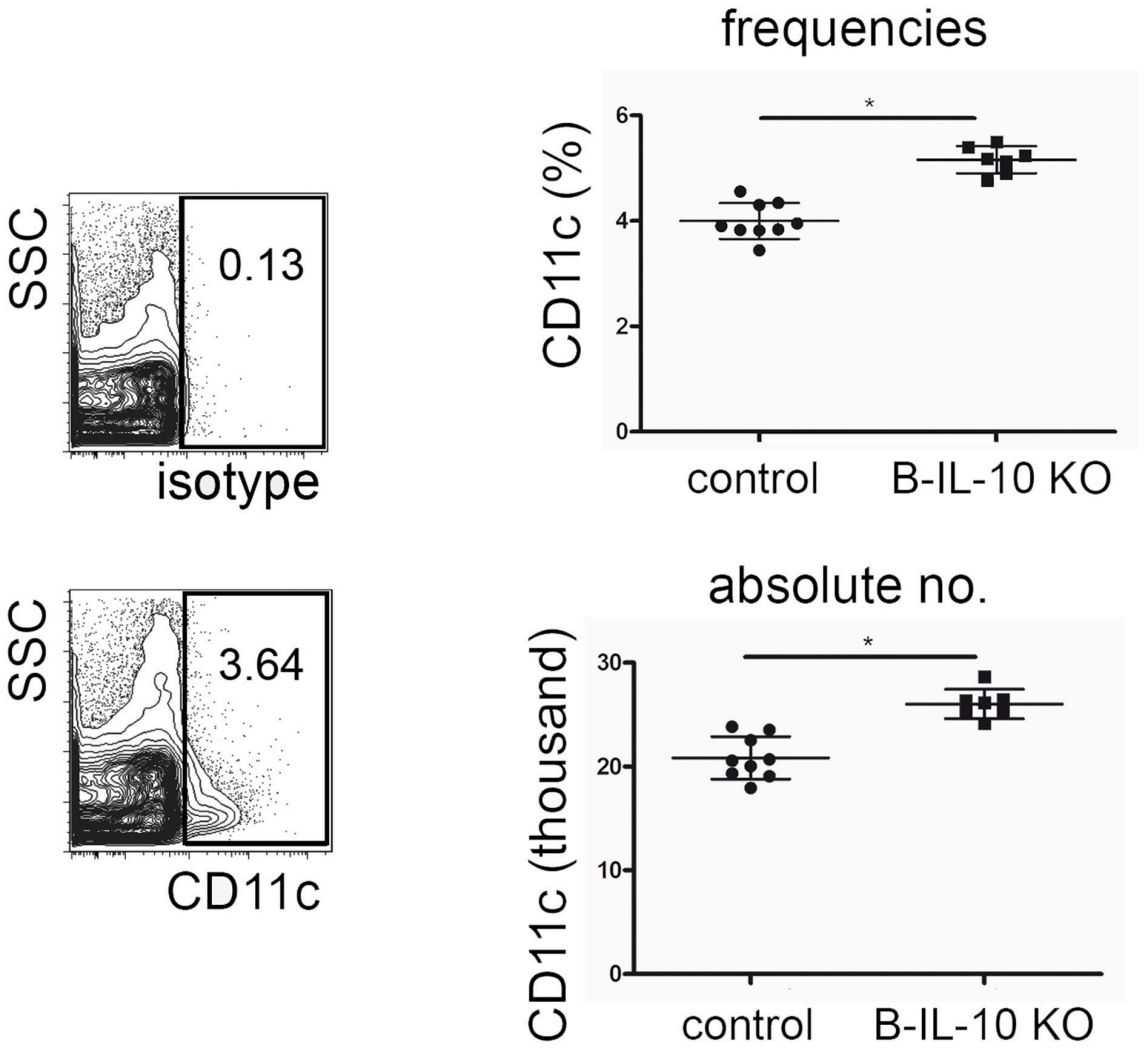
Dendritic cell populations are expanded in the bone marrow of old B lineage deficient IL-10 KO mice. Single cell suspensions from femurs and tibia of 7 months old B cell specific IL-10 KO mice and their littermate controls were stained for cell lineage markers and analyzed by flow cytometry. Representative CD11c staining and isotype control (left). Frequencies (upper panel) and absolute numbers (lower panel) of CD11+ cells in IL-10 KO mice and controls, as indicated. Pooled data from two experiments are shown. Each dot represents data from a single mouse. Statistics: *t*-test. ^*^P < 0.05.

In accordance with the expected IL-10 mediated suppression of dendritic cell formation observed *in vitro*, aged B cell–specific IL-10 knockout mice showed increased frequencies and numbers of CD11c+ dendritic cells in the bone marrow (Figure 8). Younger B cell–specific IL-10 knockout mice of 10 to 14 weeks of age, did not show differences in F4/80+ or CD11c+ cell populations compared to age matched wild type mice (Figure S2). Together, these data suggest that plasma cell derived IL-10 exhibits a non-redundant effect on local myeloid differentiation, and that this effect increases with age.

**Figure 9:**
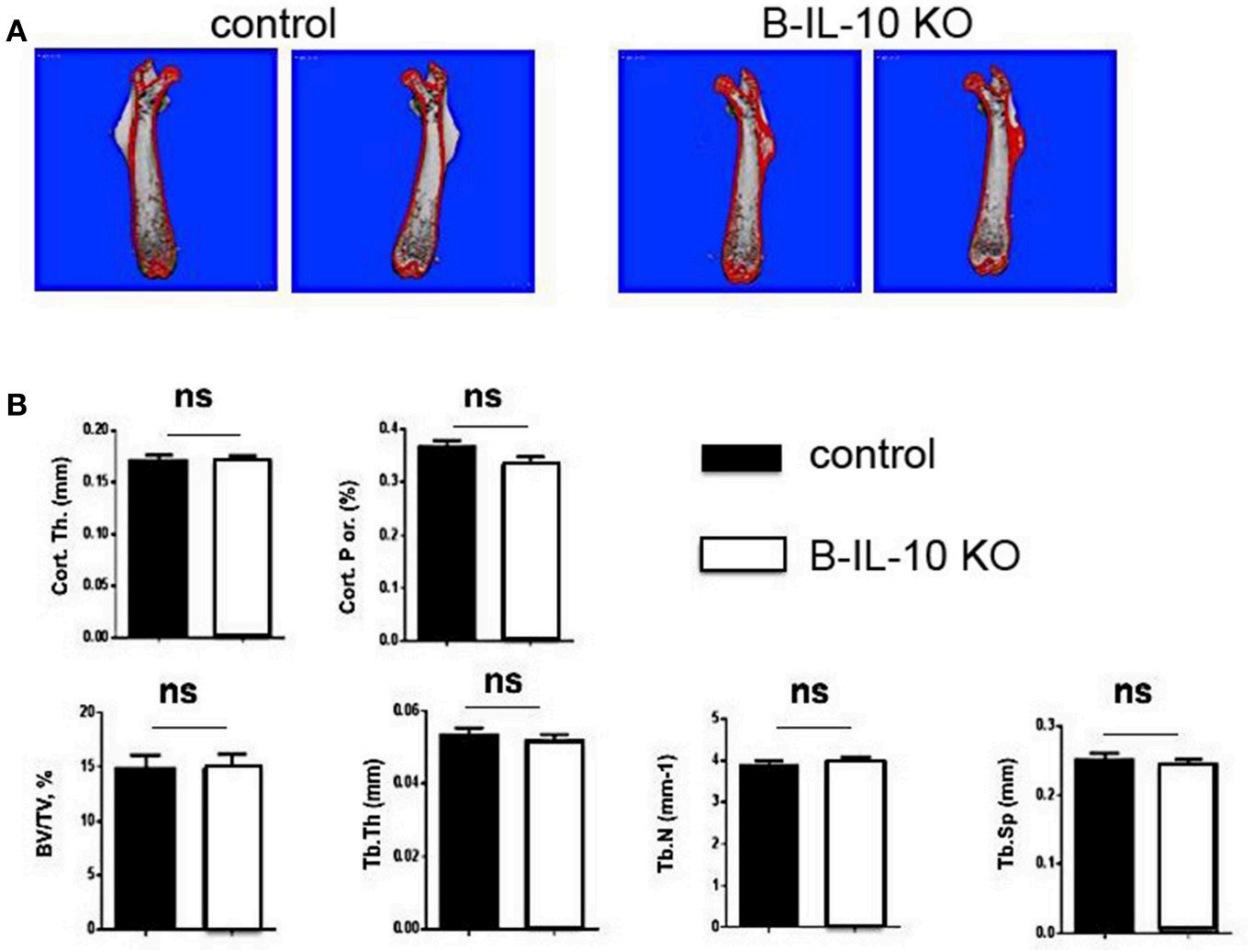
B cell deficient IL-10 KO mice exhibit normal bone formation. Femurs were dissected from B cell specific IL-10 deficient mice and littermate controls and the bone thickness and bone volume density (BV/TV) was analyzed. **(A)** Imaging of the femurs by μCT. **(B)** Statistical analysis of BV/TV. B-IL-10 KO = B cell specific IL-10 deficient mice; control = littermate controls. Statistics: *t*-test (*n* = 10 and 19 for B cell specific IL-10 deficient mice and littermate controls, respectively).

In order to investigate possible changes of the architecture of bones, the skeletal structures of femurs of 5 months old B cell–specific IL-10 knockout mice and littermate controls were analyzed by micro-computed tomography (micro-CT). No differences were observed between the volume and structure of bones of the two mouse lines (Figures 9A,B), suggesting that plasma cell derived IL-10 does not play a non-redundant function in bone homeostasis. However, this result could not rule out the possibility that plasma cell derived IL-10 becomes a relevant modulator of osteoclastogenesis and bone constitution at an even later age, and/or that IL-10 from non-plasma cells exhibits redundant functions.

The changes in CD11c+, F4/80+, and CD115+ myeloid populations in the bone marrow of aged B cell–specific IL-10 knockout mice correspond well with the effects of IL-10 from B cell/plasma cells observed in our co-culture experiments. The finding that ~90% of IL-10+ B lineage cells in bone marrow were CD138+ plasma cells implies that plasma cell derived IL-10 modulates local myeloid differentiation in the bone marrow.

In conclusion, IL-10 from bone marrow plasma cells affects CD115+ myeloid cells and DC and macrophage differentiation in an age-dependent manner, while it may have no non-redundant impact on bone homeostasis.

## Discussion

The findings presented in this paper indicate that bone marrow plasma cells provide an important local source for IL-10 that is relevant for normal formation and differentiation of myeloid populations in older mice already under homeostatic conditions. Previous reports on IL-10+ CD138+ plasmablasts and plasma cells in LN and spleen indicate that these cells are formed in the course of autoimmune diseases or infections and exhibit the capacity to limit the inflammatory reaction accompanying these conditions (36, 38, 40). In contrast, bone marrow IL-10+ plasma cells are already present and relevant under steady state conditions. Though a population of “natural regulatory plasma cells” that exhibit the capacity to rapidly up-regulate IL-10 expression has been detected in spleens of naïve mice, these cells require additional signals provided via a Toll-like receptor (TLR)-driven mechanism to up-regulate IL-10 production (40). In contrast, isolated bone marrow plasma cells described in this study secreted IL-10 into the culture supernatant without TLR-stimulation, suggesting that these cells constitutively produce IL-10. Moreover, in IL-10 transcriptional reporter mice, these plasma cells were identified by eGFP expression, again suggesting that they already produce IL-10 in naïve mice. Therefore, we assume that IL-10+ bone marrow plasma cells are not related to IL-10+ plasmablasts/plasma cells induced in peripheral lymphoid tissues in response to external or auto-inflammatory stimulation. Hence, IL-10 is produced by a specialized subset of “natural regulatory plasma cells” relevant during conditions of infection (40), but is also produced by a considerable proportion of mature bone marrow plasma cells that seem to modulate myeloid cells under steady state. Our data are in line with previous reports showing that IL-10 is a potent modulator of monocytes/macrophage differentiation and osteoclast formation. In particular, this cytokine has been shown to inhibit the differentiation of GM-CSF stimulated human monocytes into CD1a+ and MHC II high DCs, but instead promoted the formation of macrophages (25). Our results show that IL-10 exhibits similar effects on murine myeloid cell cultures and that bone marrow of aged B lineage specific IL-10 KO mice contains larger monocyte populations but smaller macrophage populations. In these mice, plasma cells represent the dominant source of IL-10 within B lineage cells. Together, these data indicate that bone marrow plasma cell derived IL-10 modulates the formation of monocytes and macrophages during hematopoiesis.

In mice infected with cecal bacteria, IL-10 has been shown to be essential for the differentiation of monocytes into a particular population of MHC II(lo) macrophages that efficiently can phagocytose apoptotic cells (26). In accordance with this observation, another study has shown that IL-10 constrains inflammation-induced macrophage phagocytosis of healthy self-cells (45). Recent findings also showed that the anti-inflammatory effects of IL-10 on macrophages are mediated by a metabolic reprograming of those cells and by eliminating their dysfunctional mitochondria (46). In murine sepsis, IL-10 has been reported to suppress the expression of IL-27 by activated F4/80+CD11b+ macrophages in an STAT3-dependent pathway (47). In accordance with a central role of IL-10 mediated signals on macrophage differentiation, mice exhibiting macrophage-specific IL-10R deficiency have been demonstrated to show impaired conditioning of monocyte-derived macrophages resulting in spontaneous development of severe colitis (48). Moreover, IL-10 seems also to play a critical role in regulating the switch of muscle macrophages from an M1 to an M2 phenotype in injured muscles *in vivo* (49). Though the importance of IL-10 mediated signals for monocyte/macrophage differentiation is well documented, most studies neither have identified the relevant cellular sources nor have addressed the question of whether the effect of IL-10 is age-dependent.

The effects of bone marrow plasma cell derived IL-10 on myeloid lineage cells observed in the present study apparently increase with age. This finding is in line with an overwhelming amount of literature showing that aging is accompanied by several changes of the immune system leading to increased vulnerability of older individuals to infectious diseases and reduced response to vaccination (31, 50–53). Among the factors associated with age-related changes of immune functions is IL-10. Our data suggest that the accumulation of IL-10+ plasma cells within the bone marrow contributes to the aging of the immune system and the related immune dysfunctions often observed in older individuals.

In aged mice, mononuclear myeloid cells have been reported to suppress the production of innate inflammatory cytokines (29). Impaired proliferation of aged human peripheral blood mononuclear cells was found to be related with increased IL-10 production (31). Moreover, human T cells exhibit age-related changes in their capacity to produce IL-10 following re-stimulation (32). IL-10 production has been reported to be oppositely affected during aging in different rat strains (30), hence indicating the existence of a genetic component influencing age-related changes in IL-10 functions. The impact of bone marrow plasma cell derived IL-10 on myeloid cells described in this study needs to be elucidated regarding the development of clinically relevant age-related immunodeficiency.

In addition to their crucial role for immune protection, monocytes, macrophages and dendritic cells play multiple roles in other physiological and pathological processes, such as wound healing, autoimmune inflammation and atherosclerosis (54–58). Further studies are required to investigate the impact of plasma cell derived IL-10 on myeloid lineage differentiation in the bone marrow, and its contribution to the age-related changes observed on these processes.

## Methods

### Mice

8-12 week-old C57BL/6 mice were purchased from Charles River Laboratories (Sulzfeld, Germany). IL-10 reporter (Vert-X) mice were provided by Prof. Christopher L. Karp (University of Cincinnati College of Medicine, Cincinnati, Ohio, US), and CD19 Cre and IL-10 flox/flox mice were provided by Dr. Axel Roers, Dresden, Germany. Mice were kept and experiments were performed at the animal facilities of the University of Luebeck. All of the procedures performed for research purposes were approved by the governmental administration of the state of Schleswig-Holstein, Germany.

### Antibodies

Anti-mouse antibodies used in flow cytometry staining analysis: anti-CD11b (clone M1/70.15.11, in house production); anti-CD19 (clone 1D3, BioLegend, Fell, Germany); anti-CD138 (clone 281-2, BioLegend); anti-CD210 (IL-10 receptor, clone 1B1.3, in house production); F4/80 (clone BM8, in house production): CD115 (clone AFS98, BioLegend); anti-B220 (clone RA3.B2, in house production); anti-GR1 (clone RB6-8C5, in house production); anti-Ly6G (clone 1A8, BioLegend); anti-CD4 (clone GK1.5, eBioscience, Frankfurt, Germany); Ly-6C (clone HK1.4, BioLegend), CD11c (clone N418, Biolegend).

### Flow Cytometry

Single cell suspension of spleens and bone marrow (femurs and tibia) were prepared and filtered through a 70 μm cell strainer (BD Falcon). The primary cells were resuspended (10^7^ cells/ml) in PBS containing 0.5%. BSA 0.5%. *In vitro* generated CD115+ cells were generated as described below. To harvest them, culture supernatants were removed and cells were incubated with 2 mM EDTA for 10 min, and then harvested with cell scrapers.

Fc receptors were blocked with anti-CD16/CD32 for 15 min (5 μg/ml in PBS/BSA, clone 2.4G2, in house production). Subsequently, cells were washed with ice-cold PBS/0.5%BSA, and incubated with fluorescent labeled antibodies for 10 min on ice. After washing twice, cells were re-suspended in PBS/0.5% BSA/2 mM EDTA, and analyzed in an LSRII flow cytometer (BD Biosciences). The resulting data were analyzed using the FlowJo software.

### Myeloid Cell Cultures

In order to generate sufficient numbers of CD115+ cells, primary bone marrow were cultured (250 × 10^3^ cell / well) in 48-well plates for 2 days in RPMI1640 (Gibco) medium containing 30 ng/ml M-CSF (R&D). After 2 days, non-adherent cells were removed and the purity of CD115+ cells was determined by flow cytometry.

In order to stimulate the expansion and further differentiation of these CD115+ cells into DCs or macrophages, at day 0 the culture was supplemented with 30 ng/ml M-CSF and 50 ng/ml RANKL (R&D) in RPMI1640 (Gibco). After 2 days of culture alone, or together with recombinant IL-10 or activated B cells/plasma cells, cells were harvested and analyzed by flow cytometry.

To generate osteoclast-like cells, cultures of CD115+ cells were prolonged for up to seven days. These cultures were supplemented with 30 ng/ml M-CSF and 50 ng/ml RANKL at days 0 and 3 in RPMI1640 (Gibco). In order to quantify osteoclast like cells, Tartrate-resistant acid phosphatase (TRAP) staining was performed with a commercial kit (Sigma-Aldrich). Briefly, cells were washed twice with cold PBS to remove non-adherent cells, and then fixed with 250 μl 4% PFA for 3 min. After washing twice with PBS, 250 μl TRAP solution was added and incubated for 15 min. Samples were washed again two times and mounted with glycerin/PBS (1:1). Purple cells with ≥3 nuclei were quantified as osteoclasts by microscope.

### B Cell and Plasma Cell Isolation and Culture

For the co-culture experiments, B cells were isolated from spleen using a MACS B cell isolation kit (Miltenyi Biotech, Berdgisch Gladbach, Germany). The isolated B cells were incubated in complete RPMI medium with 10 μg/ml LPS (Sigma-Aldrich) in 48 well plates. For the measurement of IL-10 in the supernatant of cultures, CD138+ plasma cells, CD138− CD19+ B cells and CD138− CD19− non B cells from bone marrow were sorted by FACS using a MoFlo Legacy (Beckman Coulter) and cultured in 98 well plates (20 thousand/cells well) in complete RPMI medium plus 20 ng/ml IL-6. This cytokine was added because it improved the survival of isolated plasma cells, which otherwise die very quickly.

### IL-10 ELISA

IL-10 in the supernatant of the murine B cell/plasma cell cultures was measured using a mouse IL-10 ELISA kit (Biolegend) according to the instructions of the manufacturer.

### RNA Isolation and Real Time PCR

Bone marrow plasma cells (CD138+), non-plasma cells (CD138−), dendritic cells (CD115+ Ly6C− CD11c+), neutrophils (CD115− Ly6C− CD11b+ Ly6G+), macrophages (CD115− CD11c− F4/80+) and monocytes (CD115+ Ly6C+ CD11c−) were sorted using a BD FACS ARIA III. RNA was isolated using Trizol reagent according to the manufacturer's instructions (Zymo Research). Reverse transcription reaction of total RNA was performed using a QuantiNova Reverse Transcription Kit (Qiagen), including the procedure for removal of contaminating genomic DNA, according to manufacturer's instructions. Quantitative PCR was done using QuantiNova SYBR Green PCR Kit (Qiagen) iQSyber green (Biorad) on a CFX96 real-time PCR system (Biorad) using the specific following primers (Metabion): IL-10 forward 5′-GCGCTGTCATCGATTTCTCC-3′ and reverse 5′-GGCCTTGTAGACACCTTGGTC-3′; IL-10RA forward 5′- GAGCCTAGAATTCATTGCATACG-3′ and reverse 5′-GTACTGTTTGAGGGCCACTT-3′; actin forward 5′-GCACCACACCTTCTACAATGAG-3′ and reverse 5′-AAATAGCACAGCCTGGATAGCAAC-3′ (used as internal control for all samples). Real-time RT-PCR data were analyzed using CFX Manager Software 3.1 (Bio-Rad).

### μCT Scanning

μCT analysis of the fixed femur was performed using a μCT40 desktop cone-beam microCT (Scanco Medical, Switzerland) with a voxel size of 10 μm. Thereby, trabecular bone was evaluated in the distal metaphysis in a volume situated 2,100 μm to 600 μm proximal of the distal growth plate. Cortical bone was evaluated in a volume of 1,000 μm length situated in the middle of the diaphysis.

### Statistics

Statistical calculations were performed using GraphPad Prism (GraphPad Software, La Jolla, USA). ^*^P < 0.05, ^**^P < 0.01, ^***^P < 0.001. Statistical tests are indicated in the individual figure legends.

## Ethics Statement

The animal experiments conducted in this study were done in strict accordance with the German regulations of the Society for Laboratory Animal Science (GVSOLAS), and the European Health Law of the Federation of Laboratory Animal Science Associations (FELASA). All animal experiments were approved by the respective local Committee on the Ethics of Animal Experiments of the state Schleswig-Holstein (Ministerium für Landwirtschaft, Umwelt und ländliche Räume des Landes Schleswig Holstein).

## Author Contributions

LM, LA, A-KC, TL, JL, CL, KH, UK, and DW performed experiments. LM, LA, J-PD, and RM designed the experiments and wrote the manuscript.

### Conflict of Interest Statement

The authors declare that the research was conducted in the absence of any commercial or financial relationships that could be construed as a potential conflict of interest.

## References

[1] Haaijman JJ Schuit HR Hijmans W. Immunoglobulin-containing cells in different lymphoid organs of the CBA mouse during its life-span. Immunology. (1977) 32:427–34. 608677 PMC1445506

[2] Slifka MK Matloubian M Ahmed R. Bone marrow is a major site of long-term antibody production after acute viral infection. J Virol. (1995) 69:1895–902. 7853531 10.1128/jvi.69.3.1895-1902.1995 PMC188803

[3] Manz RA Hauser AE Hiepe F Radbruch A. Maintenance of serum antibody levels. Annu Rev Immunol. (2005) 23:367–86. 10.1146/annurev.immunol.23.021704.115723 15771575

[4] Tew JG DiLosa RM Burton GF Kosco MH Kupp LI Masuda A. Germinal centers and antibody production in bone marrow. Immunol Rev. (1992) 126:99–112. 10.1111/j.1600-065X.1992.tb00633.x 1597323

[5] Blanc P Moro-Sibilot L Barthly L Jagot F This S de Bernard S. Mature IgM-expressing plasma cells sense antigen and develop competence for cytokine production upon antigenic challenge. Nat Commun. (2016) 7:13600. 10.1038/ncomms13600 27924814 PMC5150646

[6] Rodríguez-Bayona B Ramos-Amaya A López-Blanco R Campos-Caro A Brieva JA. STAT-3 activation by differential cytokines is critical for human *in vivo*-generated plasma cell survival and Ig secretion. J Immunol. (2013) 191:4996–5004. 10.4049/jimmunol.1301559 24101550

[7] Medina F Segundo C Campos-Caro A González-García I Brieva JA. The heterogeneity shown by human plasma cells from tonsil, blood, and bone marrow reveals graded stages of increasing maturity, but local profiles of adhesion molecule expression. Blood. (2002) 99:2154–61. 10.1182/blood.V99.6.2154 11877292

[8] Mumtaz IM Hoyer BF Panne D Moser K Winter O Cheng QY. Bone marrow of NZB/W mice is the major site for plasma cells resistant to dexamethasone and cyclophosphamide: implications for the treatment of autoimmunity. J Autoimmun. (2012) 39:180–8. 10.1016/j.jaut.2012.05.010 22727274

[9] Prosper F Verfaillie CM. Regulation of hematopoiesis through adhesion receptors. J Leukoc Biol. (2001) 69:307–16. 10.1189/jlb.69.3.307 11261776

[10] Shen Y Nilsson SK. Bone, microenvironment and hematopoiesis. Curr Opin Hematol. (2012) 19:250–5. 10.1097/MOH.0b013e328353c714 22504524

[11] Yona S Jung S. Monocytes: subsets, origins, fates and functions. Curr Opin Hematol. (2010) 17:53–9. 10.1097/MOH.0b013e3283324f80 19770654

[12] Xiao Y Palomero J Grabowska J Wang L de Rink I van Helvert L. Macrophages and osteoclasts stem from a bipotent progenitor downstream of a macrophage/osteoclast/dendritic cell progenitor. Blood Adv. (2017) 1:1993–2006. 10.1182/bloodadvances.2017008540 29296846 PMC5728283

[13] Ludin A Itkin T Gur-Cohen S Mildner A Shezen E Golan K. Monocytes-macrophages that express α-smooth muscle actin preserve primitive hematopoietic cells in the bone marrow. Nat Immunol. (2012) 13:1072–82. 10.1038/ni.2408 22983360

[14] Mildner A Marinkovic G Jung S Murine monocytes: origins, subsets, fates, and functions. Microbiol Spectr. (2016) 1–9 4 10.1128/microbiolspec.MCHD-0033-2016 27780020

[15] Koelwyn GJ Corr EM Erbay E Moore KJ. Regulation of macrophage immunometabolism in atherosclerosis. Nat Immunol. (2018) 19:526–37. 10.1038/s41590-018-0113-3 29777212 PMC6314674

[16] Shi C Pamer EG. Monocyte recruitment during infection and inflammation. Nat Rev Immunol. (2011) 11:762–74. 10.1038/nri3070 21984070 PMC3947780

[17] Jakubzick CV Randolph GJ Henson PM. Monocyte differentiation and antigen-presenting functions. Nat Rev Immunol. (2017) 17:349–62. 10.1038/nri.2017.28 28436425

[18] Wynn TA Chawla A Pollard JW. Macrophage biology in development, homeostasis and disease. Nature. (2013) 496:445–55. 10.1038/nature12034 23619691 PMC3725458

[19] Swiecki M Colonna M. Unraveling the functions of plasmacytoid dendritic cells during viral infections, autoimmunity, and tolerance. Immunol Rev. (2010) 234:142–62. 10.1111/j.0105-2896.2009.00881.x 20193017 PMC3507434

[20] Takeshita S Faccio R Chappel J Zheng L Feng X Weber JD. c-Fms tyrosine 559 is a major mediator of M-CSF-induced proliferation of primary macrophages. J Biol Chem. (2007) 282:18980–90. 10.1074/jbc.M610938200 17420255

[21] Lutz MB Strobl H Schuler G Romani N. GM-CSF monocyte-derived cells and langerhans cells as part of the dendritic cell family. Front Immunol. (2017) 8:1388. 10.3389/fimmu.2017.01388 29109731 PMC5660299

[22] Ushach I Zlotnik A. Biological role of granulocyte macrophage colony-stimulating factor (GM-CSF) and macrophage colony-stimulating factor (M-CSF) on cells of the myeloid lineage. J Leukoc Biol. (2016) 100:481–9. 10.1189/jlb.3RU0316-144R 27354413 PMC4982611

[23] Mocellin S Panelli MC Wang E Nagorsen D Marincola FM. The dual role of IL-10. Trends Immunol. (2003) 24:36–43. 10.1016/S1471-4906(02)00009-1 12495723

[24] Ouyang W Rutz S Crellin NK Valdez PA Hymowitz SG. Regulation and functions of the IL-10 family of cytokines in inflammation and disease. Annu Rev Immunol. (2011) 29:71–109. 10.1146/annurev-immunol-031210-101312 21166540

[25] Allavena P Piemonti L Longoni D Bernasconi S Stoppacciaro A Ruco L. IL-10 prevents the differentiation of monocytes to dendritic cells but promotes their maturation to macrophages. Eur J Immunol. (1998) 28:359–69. 10.1002/(SICI)1521-4141(199801)28:01<359::AID-IMMU359>3.0.CO;2-4 9485215

[26] Nguyen H-H Tran B-T Muller W Jack RS. IL-10 acts as a developmental switch guiding monocyte differentiation to macrophages during a murine peritoneal infection. J Immunol. (2012) 189:3112–20. 10.4049/jimmunol.1200360 22869902

[27] Martin C Espaillat MP Santiago-Schwarz F. IL-10 restricts dendritic cell (DC) growth at the monocyte-to-monocyte-derived DC interface by disrupting anti-apoptotic and cytoprotective autophagic molecular machinery. Immunol Res. (2015) 63:131–43. 10.1007/s12026-015-8700-y 26395023

[28] Minciullo PL Catalano A Mandraffino G Casciaro M Crucitti A Maltese G. Inflammaging and anti-inflammaging: the role of cytokines in extreme longevity. Arch Immunol Ther Exp. (2016) 64:111–26. 10.1007/s00005-015-0377-3 26658771

[29] Chiu B-C Stolberg VR Chensue SW. Mononuclear phagocyte-derived IL-10 suppresses the innate IL-12/IFN-gamma axis in lung-challenged aged mice. J Immunol. (2008) 181:3156–66. 10.4049/jimmunol.181.5.3156 18713986

[30] Dimitrijević M Stanojević S Vujić V Aleksić I Pilipović I Leposavić G. Aging oppositely affects TNF-α and IL-10 production by macrophages from different rat strains. Biogerontology. (2014) 15:475–86. 10.1007/s10522-014-9513-4 25009084

[31] Castle SC Uyemura K Crawford W Wong W Klaustermeyer WB Makinodan T. Age-related impaired proliferation of peripheral blood mononuclear cells is associated with an increase in both IL-10 and IL-12. Exp Gerontol. (1999) 34:243–52. 10.1016/S0531-5565(98)00064-3 10363790

[32] Gardner EM Murasko DM. Age-related changes in Type 1 and Type 2 cytokine production in humans. Biogerontology. (2002) 3:271–90. 10.1023/A:1020151401826 12237564

[33] Madan R Demircik F Surianarayanan S Allen JL Divanovic S Trompette A. Nonredundant roles for B cell-derived IL-10 in immune counter-regulation. J Immunol. (2009) 183:2312–20. 10.4049/jimmunol.0900185 19620304 PMC2772089

[34] Fillatreau S Sweenie CH McGeachy MJ Gray D Anderton SM. B cells regulate autoimmunity by provision of IL-10. Nat Immunol. (2002) 3:944–50. 10.1038/ni833 12244307

[35] Shen P Roch T Lampropoulou V O'Connor RA Stervbo U Hilgenberg E. IL-35-producing B cells are critical regulators of immunity during autoimmune and infectious diseases. Nature. (2014) 507:366–70. 10.1038/nature12979 24572363 PMC4260166

[36] Matsumoto M Baba A Yokota T Nishikawa H Ohkawa Y Kayama H. Interleukin-10-producing plasmablasts exert regulatory function in autoimmune inflammation. Immunity. (2014) 41:1040–51. 10.1016/j.immuni.2014.10.016 25484301

[37] Hofmann K Clauder A-K Manz RA. Targeting B cells and plasma cells in autoimmune diseases. Front Immunol. (2018) 9:835 10.3389/fimmu.2018.00835 29740441 PMC5924791

[38] Kulkarni U Karsten CM Kohler T Hammerschmidt S Bommert K Tiburzy B. IL-10 mediates plasmacytosis-associated immunodeficiency by inhibiting complement-mediated neutrophil migration. J Allergy Clin Immunol. (2016) 137:1487–97.e6. 10.1016/j.jaci.2015.10.018 26653800

[39] Srikakulapu P Hu D Yin C Mohanta SK Bontha SV Peng L Artery tertiary lymphoid organs control multilayered territorialized atherosclerosis B-cell responses in aged ApoE-/- mice. Arterioscler Thromb Vasc Biol. (2016) 36:1174–85. 10.1161/ATVBAHA.115.306983 27102965 PMC4894775

[40] Lino AC Dang VD Lampropoulou V Welle A Joedicke J Pohar J. LAG-3 inhibitory receptor expression identifies immunosuppressive natural regulatory plasma cells. Immunity. (2018) 49:120–33. 10.1016/j.immuni.2018.06.007 30005826 PMC6057275

[41] Suzuki-Yamazaki N Yanobu-Takanashi R Okamura T Takaki S. IL-10 production in murine IgM+ CD138hi cells is driven by Blimp-1 and downregulated in class-switched cells. Eur J Immunol. (2017) 47:493–503. 10.1002/eji.201646549 28012163

[42] Park-Min K-H Ji J-D Antoniv T Reid AC Silver RB Humphrey MB. IL-10 suppresses calcium-mediated costimulation of receptor activator NF-kappa B signaling during human osteoclast differentiation by inhibiting TREM-2 expression. J Immunol. (2009) 183:2444–55. 10.4049/jimmunol.0804165 19625651 PMC2742169

[43] Amarasekara DS Yun H Kim S Lee N Kim H Rho J. Regulation of osteoclast differentiation by cytokine networks. Immune Netw. (2018) 18:e8. 10.4110/in.2018.18.e8 29503739 PMC5833125

[44] Winkler IG Sims NA Pettit AR Barbier V Nowlan B Helwani F. Bone marrow macrophages maintain hematopoietic stem cell (HSC) niches and their depletion mobilizes HSCs. Blood. (2010) 116:4815–28. 10.1182/blood-2009-11-253534 20713966

[45] Bian Z Shi L Guo Y-L Lv Z Tang C Niu S. Cd47-Sirpα interaction and IL-10 constrain inflammation-induced macrophage phagocytosis of healthy self-cells. Proc Natl Acad Sci USA. (2016) 113:E5434–43. 10.1073/pnas.1521069113 27578867 PMC5027463

[46] Ip WKE Hoshi N Shouval DS Snapper S Medzhitov R. Anti-inflammatory effect of IL-10 mediated by metabolic reprogramming of macrophages. Science. (2017) 356:513–9. 10.1126/science.aal3535 28473584 PMC6260791

[47] Bosmann M Russkamp NF Strobl B Roewe J Balouzian L Pache F Interruption of macrophage-derived IL-27(p28) production by IL-10 during sepsis requires STAT3 but not SOCS3. J Immunol. (2014) 193:5668–77. 10.4049/jimmunol.1302280 25348624 PMC4239188

[48] Zigmond E Bernshtein B Friedlander G Walker CR Yona S Kim K-W Macrophage-restricted interleukin-10 receptor deficiency, but not IL-10 deficiency, causes severe spontaneous colitis. Immunity. (2014) 40:720–33. 10.1016/j.immuni.2014.03.012 24792913

[49] Deng B Wehling-Henricks M Villalta SA Wang Y Tidball JG. IL-10 triggers changes in macrophage phenotype that promote muscle growth and regeneration. J Immunol. (2012) 189:3669–80. 10.4049/jimmunol.1103180 22933625 PMC3448810

[50] Pallikkuth S de Armas L Rinaldi S Pahwa S. T follicular helper cells and B cell dysfunction in aging and HIV-1 infection. Front Immunol. (2017) 8:1380. 10.3389/fimmu.2017.01380 29109730 PMC5660291

[51] Jergović M Smithey MJ Nikolich-Žugich J. Intrinsic and extrinsic contributors to defective CD8+ T cell responses with aging. Exp Gerontol. (2018) 105:140–5. 10.1016/j.exger.2018.01.011 29337070

[52] El Chakhtoura NG Bonomo RA Jump RLP. Influence of aging and environment on presentation of infection in older adults. Infect Dis Clin North Am. (2017) 31:593–608. 10.1016/j.idc.2017.07.017 29079150 PMC5846087

[53] Nikolich-Žugich J. Aging of the T cell compartment in mice and humans: from no naive expectations to foggy memories. J Immunol. (2014) 193:2622–9. 10.4049/jimmunol.1401174 25193936 PMC4157314

[54] Bajpai G Schneider C Wong N Bredemeyer A Hulsmans M Nahrendorf M. The human heart contains distinct macrophage subsets with divergent origins and functions. Nat Med. (2018) 24:1234–45. 10.1038/s41591-018-0059-x 29892064 PMC6082687

[55] Fan R Toubal A Goñi S Drareni K Huang Z Alzaid F. Loss of the co-repressor GPS2 sensitizes macrophage activation upon metabolic stress induced by obesity and type 2 diabetes. Nat Med. (2016) 22:780–91. 10.1038/nm.4114 27270589

[56] Ramkhelawon B Hennessy EJ Ménager M Ray TD Sheedy FJ Hutchison S. Netrin-1 promotes adipose tissue macrophage retention and insulin resistance in obesity. Nat Med. (2014) 20:377–84. 10.1038/nm.3467 24584118 PMC3981930

[57] Robbins CS Hilgendorf I Weber GF Theurl I Iwamoto Y Figueiredo J-L. Local proliferation dominates lesional macrophage accumulation in atherosclerosis. Nat Med. (2013) 19:1166–72. 10.1038/nm.3258 23933982 PMC3769444

[58] Krzyszczyk P Schloss R Palmer A Berthiaume F. The role of macrophages in acute and chronic wound healing and interventions to promote pro-wound healing phenotypes. Front Physiol. (2018) 9:419. 10.3389/fphys.2018.00419 29765329 PMC5938667

