# Supplementary material for: Bone Marrow Plasma Cells Modulate Local Myeloid-Lineage Differentiation via IL-10
Source: Front Immunol. 2019 May 31;10:1183. doi: 10.3389/fimmu.2019.01183 (PMC6555095; doi:10.3389/fimmu.2019.01183)
Supplement: Supplementary file 1 [file Data_Sheet_1.PDF]

Supplemental data

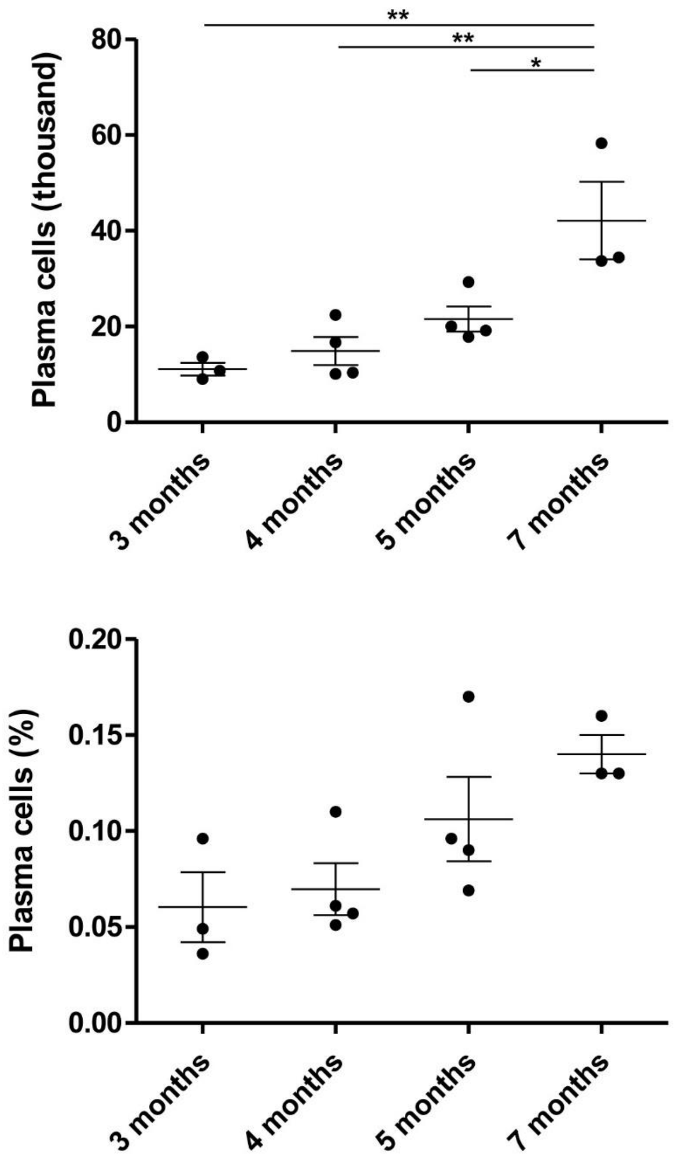

Figure S1. **Bone marrow plasma cells numbers increase with age.** CD138+/B220- bone marrow cells from femur and tibia were analyzed by flow cytometry as shown in Figure 1. Each dot represents data from one individual mouse. Representative data of one of two experiments are shown. Statistics: ANOVA.

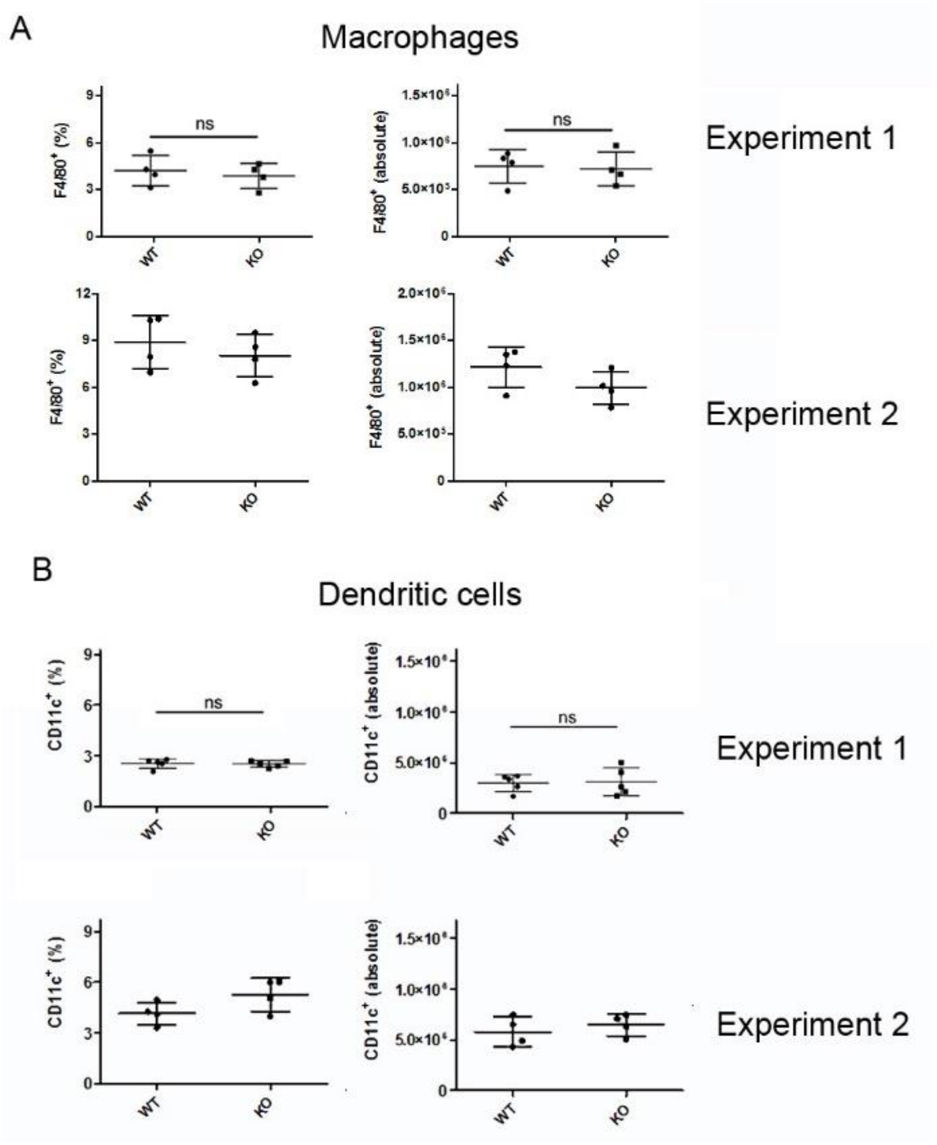

Figure S2. **Young B cell–specific IL-10 knockout mice do not show differences in bone marrow macrophage and DC populations.** Bone marrow cells from femurs and tibia of B cell–specific IL-10 knockout mice (KO) and wild type (WT) were analyzed for F4/80+/CD115- macrophage and CD11c+ DC populations as shown in Figures 7 and 8, respectively. Data from two independent experiments are shown (Experiment 1, Experiment 2). Each dot represents data from one individual mouse. In experiment 1, mice were 10 weeks of age. In experiment 2, mice were approximately 14 weeks of age. Statistics: t-test
